# Supplementary figures and images for: Detection of genetic divergence among some wheat (Triticum aestivum L.) genotypes using molecular and biochemical indicators under salinity stress
Source: PLoS One. 2021 Mar 29;16(3):e0248890. doi: 10.1371/journal.pone.0248890 (PMC8007010; doi:10.1371/journal.pone.0248890)

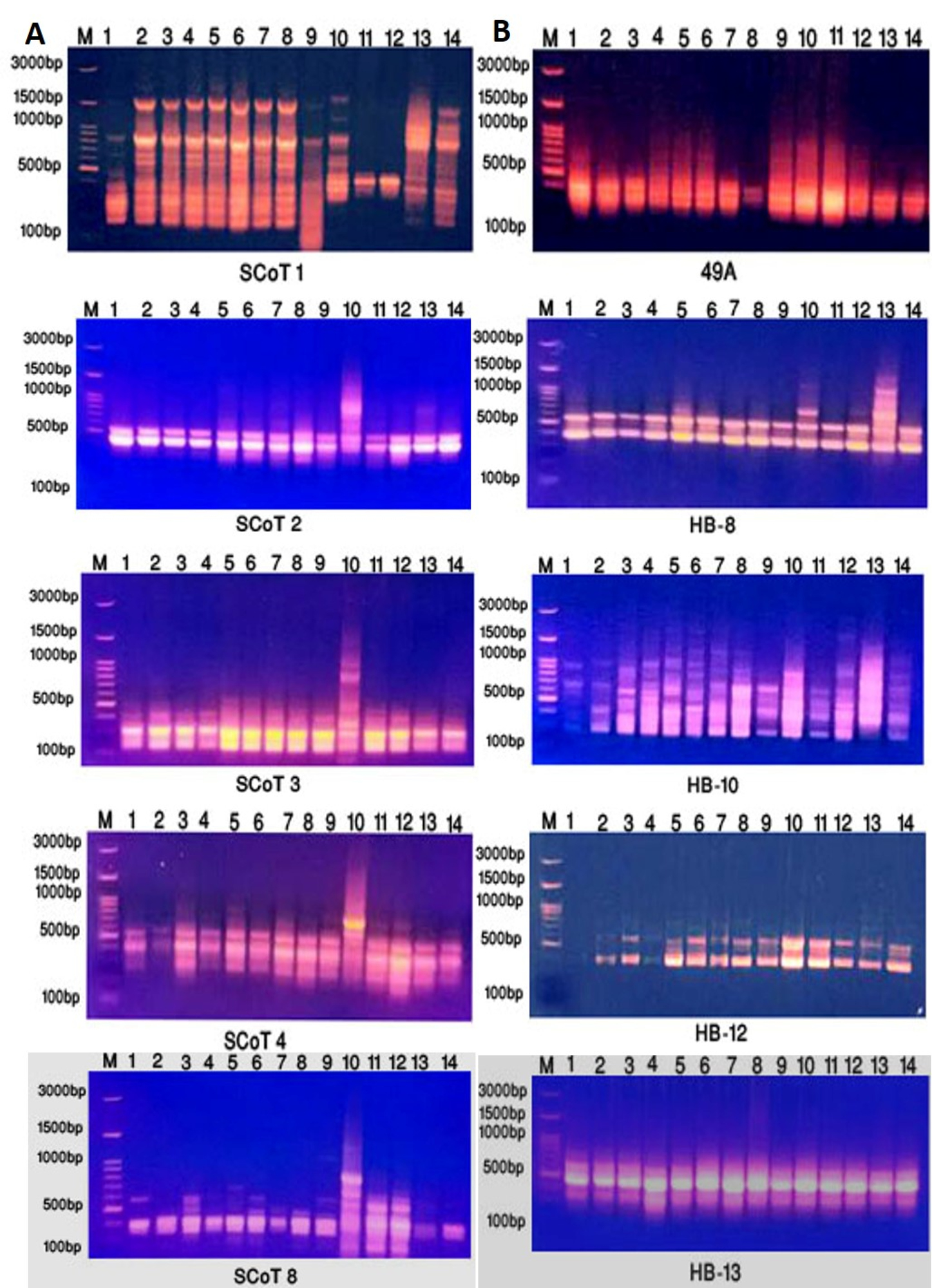

Supplement: S1 Fig — (A) SCoT and (B) ISSR fingerprinting of wheat genotypes: M; DNA marker, lanes 1–14; Misr-2, Misr-3, Sids-1, Sids-12, Sids-14, Sakha-93, Bani Suef-7, Sohag-4, Sohag-5, Shandweel-1, Sakha-95, Gemmeiza 12, Giza-168 and Misr-1. (TIF) [file pone.0248890.s001.tif]

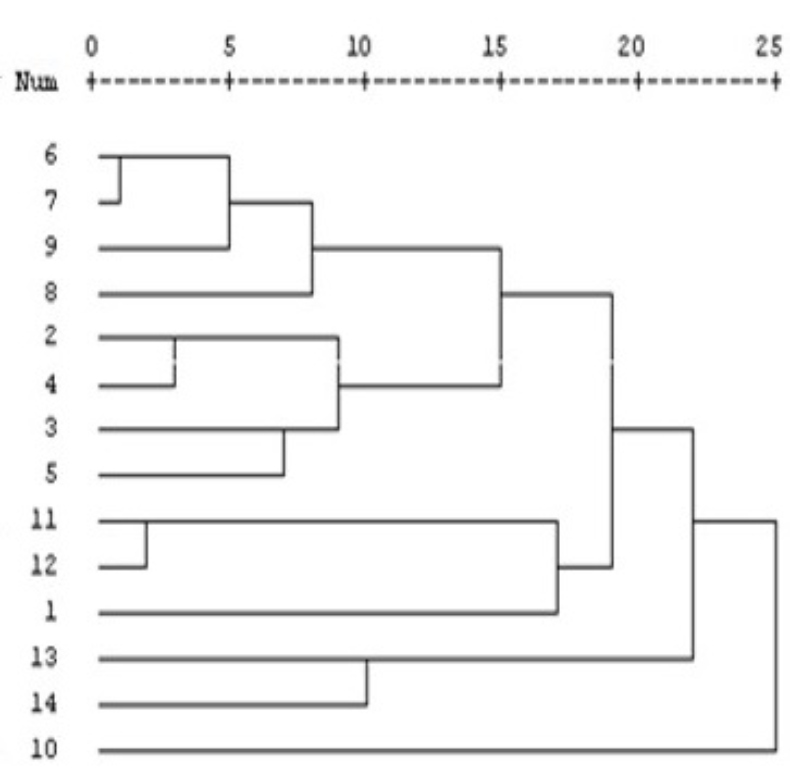

Supplement: S2 Fig — (1) Misr 2, (2) Misr 3, (3) Sids 1, (4) Sids 12, (5) Sids 14, (6) Sakha 93, (7) Bani Seuf 7, (8) Sohag 4, (9) Sohag 5, (10) Shandaweel1, (11) Sakha 95, (12) Gemmeiza 12, (13) Giza 168, and (14) Misr 1. Dendrogram calculated using Jaccard’s similarity coefficients and the UPGMA algorithm. (TIF) [file pone.0248890.s002.tif]

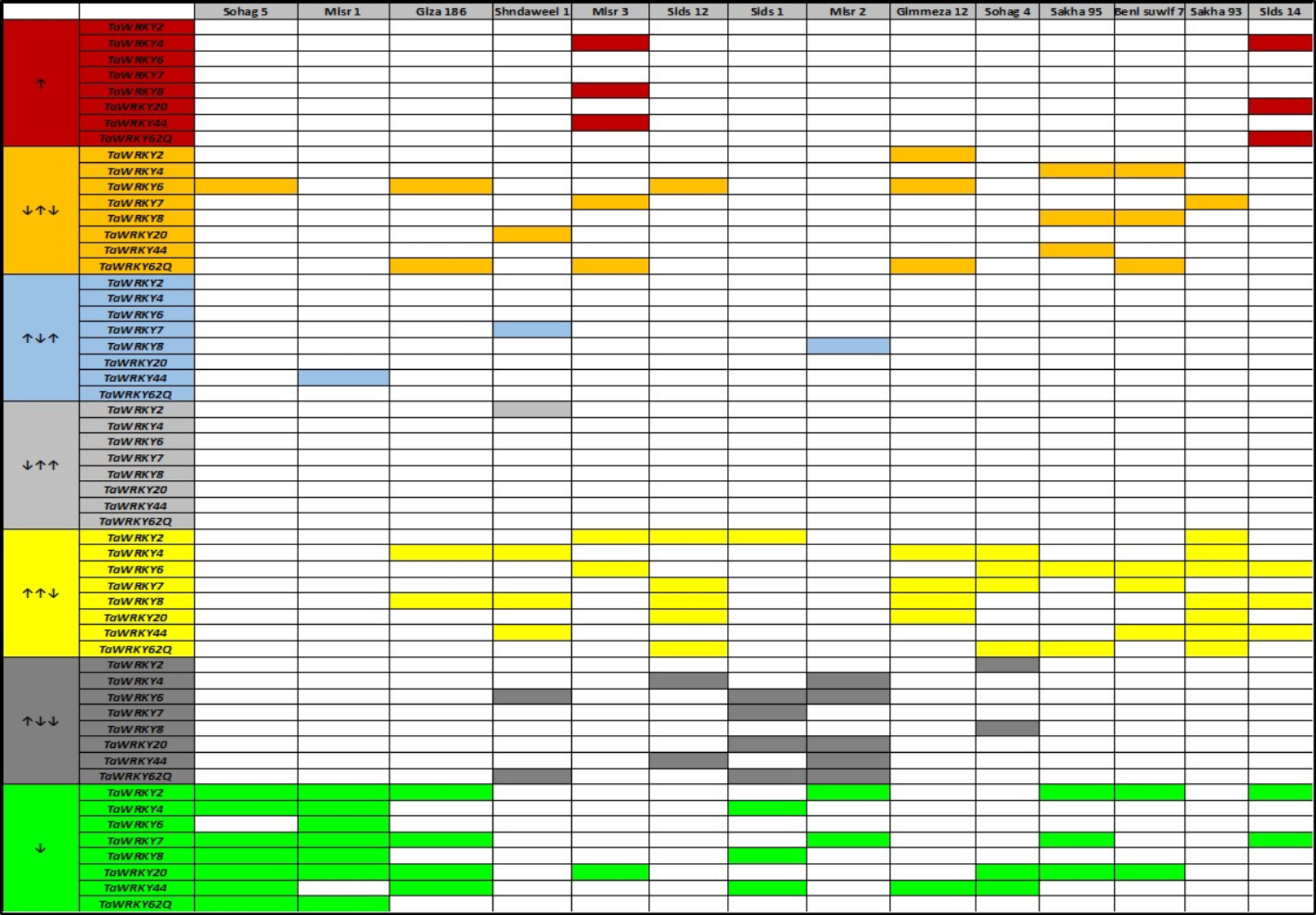

Supplement: S3 Fig — (↑) Means upregulation, (↓) means downregulation. The first pattern is coloured red and involves all the genotypes in which its expression was upregulated for all studied genes at 50, 150, and 250 mM NaCl. The second pattern is coloured orange and includes all the genotypes in which expression decreased after being subjected to 50 mM NaCl and thereafter increased after being exposed to 150 mM NaCl, ultimately being downregulated after exposure to 250 mM NaCl. The third pattern is coloured blue and includes all the genotypes; its expression was increased after exposure to 50 mM NaCl and then downregulated after exposure to 150 mM NaCl, finally being upregulated following exposure to 250 mM NaCl. The fourth pattern is coloured light grey and includes all the genotypes in which its expression decreased after exposure to 50 mM NaCl and thereafter increased after exposure to 150 and 250 mM NaCl. The fifth pattern is coloured yellow and includes all the genotypes in which its expression increased after exposure to 50 and 150 mM NaCl and then decreased after exposure to 250 mM NaCl. The sixth pattern is coloured dark grey and includes all the genotypes in which its expression increased after exposure to 50 mM NaCl and then decreased after exposure to 150 and 250 mM NaCl. The seventh pattern is coloured green and includes all the genotypes in which its expression was downregulated for the studied genes at 50, 150, and 250 mM NaCl. (TIF) [file pone.0248890.s003.tif]
