# Supplementary material for: Detection of genetic divergence among some wheat (Triticum aestivum L.) genotypes using molecular and biochemical indicators under salinity stress
Source: PLoS One. 2021 Mar 29;16(3):e0248890. doi: 10.1371/journal.pone.0248890 (PMC8007010; doi:10.1371/journal.pone.0248890)
Supplement: S1 Table — (DOCX) [file pone.0248890.s004.docx]

**S1 Table.** List of the primer names and their nucleotide sequences used in the study for the ISSR and SCoT procedure.

| **No** | **Name** | **Sequence (5→3̀)** | **No** | **Name** | **Sequence (5→3̀)** |
| --- | --- | --- | --- | --- | --- |
| 1 | **SCoT 1** | ACGACATGGCGACCACGC | 1 | **49A** | CTCTCTCTCTCTCTCTTG |
| 2 | **SCoT 2** | ACCATGGCTACCACCGGC | 2 | **HB-8** | GAGAGAGAGAGAGG |
| 3 | **SCoT 3** | ACGACATGGCGACCCACA | 3 | **HB-10** | GAGAGAGAGAGACC |
| 4 | **SCoT 4** | ACCATGGCTACCACCGCA | 4 | **HB- 12** | CACCACCACGC |
| 5 | **SCoT 8** | ACAATGGCTACCACTGAG | 5 | **HB-13** | GAGGAGGAGC |
