# Supplementary material for: Detection of genetic divergence among some wheat (Triticum aestivum L.) genotypes using molecular and biochemical indicators under salinity stress
Source: PLoS One. 2021 Mar 29;16(3):e0248890. doi: 10.1371/journal.pone.0248890 (PMC8007010; doi:10.1371/journal.pone.0248890)
Supplement: S2 Table — (DOCX) [file pone.0248890.s005.docx]

**S2 Table.** Sequences of the primers used in the real-time PCR.

| **No** | **Name** | **Primer sequence (5`→3̀)** |
| --- | --- | --- |
| 1 | *TaWRKY2* | **F** GTAACCTGGCCTGCCGCCGTGCA  **R** AGCGACTCGACGAACATGTCGT |
| 2 | *TaWRKY4* | **F** AAGAGCAGTGAGCATCCAAGGA  **R** GGCAAAGGGTGATTGTGAGAACTC |
| 3 | *TaWRKY6* | **F** CTCCGACTACTCGCCGCT  **R** CGTCGCCGTCGAACATCCC |
| 4 | *TaWRKY7* | **F** ATGGAGGAAGTACGGTCAGAA  **R** GTGCGTGCCCTCGTACGTGGTGA |
| 5 | *TaWRKY8* | **F** GTTTCGGGAACCTCCGCCTGCGC  **R** GAGTAACTGTCGGAAGGTATATC |
| 6 | *TaWRKY20* | **F** CACCACCACCACCACCTC  **R** AGCAGCGACGACGACATC |
| 7 | *TaWRKY44* | **F** CCAACGGCGGTGATAACTACAT  **R** GCTACTGGATGCTGCCTTCTG |
| 8 | *TaWRKY62Q* | **F** TCGTTGACCACCACCAG  **R** AGCCGTCCCCAAATCCA |
| 9 | *TaActin* | **F**  CTTGTATGCCAGCGGTCGAACA  **R**  CTCATAATCAAGGGCCACGTA |

(F) Forward primer; (R) reverse primer
