# Supplementary material for: Detection of genetic divergence among some wheat (Triticum aestivum L.) genotypes using molecular and biochemical indicators under salinity stress
Source: PLoS One. 2021 Mar 29;16(3):e0248890. doi: 10.1371/journal.pone.0248890 (PMC8007010; doi:10.1371/journal.pone.0248890)
Supplement: S3 Table — (DOCX) [file pone.0248890.s006.docx]

**S3 Table.** The similarity matrix based on ISSR and SCoT data.

|  | Misr 2 | Misr 3 | Sids 1 | Sids 12 | Sids 14 | Sakha 93 | Bani Seuf 7 | Sohag 4 | Sohag 5 | Shandaweel 1 | Sakha 95 | Gemmeiza 12 | Giza 168 |
| --- | --- | --- | --- | --- | --- | --- | --- | --- | --- | --- | --- | --- | --- |
| Misr 2 | 1.0 |  |  |  |  |  |  |  |  |  |  |  |  |
| Misr 3 | 0.31 | 1.0 |  |  |  |  |  |  |  |  |  |  |  |
| Sids 1 | 0.52 | 0.27 | 1.0 |  |  |  |  |  |  |  |  |  |  |
| Sids 12 | 0.50 | 0.07 | 0.16 | 1.0 |  |  |  |  |  |  |  |  |  |
| Sids 14 | 0.62 | 0.36 | 0.18 | 0.14 | 1.0 |  |  |  |  |  |  |  |  |
| Sakha 93 | 0.66 | 0.54 | 0.46 | 0.34 | 0.43 | 1.0 |  |  |  |  |  |  |  |
| Bani Seuf 7 | 0.61 | 0.54 | 0.28 | 0.10 | 0.12 | 0.00 | 1.0 |  |  |  |  |  |  |
| Sohag 4 | 0.64 | 0.52 | 0.56 | 0.54 | 0.41 | 0.16 | 0.24 | 1.0 |  |  |  |  |  |
| Sohag 5 | 0.73 | 0.61 | 0.51 | 0.49 | 0.48 | 0.10 | 0.18 | 0.21 | 1.0 |  |  |  |  |
| Shandaweel 1 | 0.97 | 0.65 | 0.96 | 0.76 | 0.74 | 0.56 | 0.52 | 0.76 | 0.62 | 1.0 |  |  |  |
| Sakha 95 | 0.59 | 0.74 | 0.51 | 0.49 | 0.36 | 0.51 | 0.46 | 0.21 | 0.14 | 0.62 | 1.0 |  |  |
| Gemmeiza 12 | 0.38 | 0.72 | 0.61 | 0.59 | 0.57 | 0.61 | 0.56 | 0.44 | 0.38 | 0.83 | 0.04 | 1.0 |  |
| Giza 168 | 0.90 | 1.00 | 0.70 | 0.80 | 0.57 | 0.49 | 0.56 | 0.48 | 0.55 | 0.78 | 0.55 | 0.75 | 1.0 |
| Misr 1 | 0.66 | 0.79 | 0.69 | 0.68 | 0.31 | 0.57 | 0.53 | 0.43 | 0.64 | 0.67 | 0.24 | 0.61 | 0.26 |
