# Supplementary material for: Detection of genetic divergence among some wheat (Triticum aestivum L.) genotypes using molecular and biochemical indicators under salinity stress
Source: PLoS One. 2021 Mar 29;16(3):e0248890. doi: 10.1371/journal.pone.0248890 (PMC8007010; doi:10.1371/journal.pone.0248890)
Supplement: S4 Table — (DOCX) [file pone.0248890.s007.docx]

**S4 Table.** Total number of genotypes/induced genes under different NaCl concentrations.

|  | ***TaWRKY2*** | ***TaWRKY4*** | ***TaWRKY6*** | ***TaWRKY7*** | ***TaWRKY8*** | ***TaWRKY20*** | ***TaWRKY44*** | ***TaWRKY62Q*** |
| --- | --- | --- | --- | --- | --- | --- | --- | --- |
| **50 mM NaCl** | **5** | **9** | **9** | **6** | **9** | **6** | **8** | **8** |
| **150 mM NaCl** | **6** | **10** | **10** | **6** | **9** | **5** | **6** | **9** |
| **250 mM NaCl** | **1** | **2** | **0** | **1** | **2** | **1** | **2** | **1** |
