# Supplementary material for: Detection of genetic divergence among some wheat (Triticum aestivum L.) genotypes using molecular and biochemical indicators under salinity stress
Source: PLoS One. 2021 Mar 29;16(3):e0248890. doi: 10.1371/journal.pone.0248890 (PMC8007010; doi:10.1371/journal.pone.0248890)
Supplement: S5 Table — (DOCX) [file pone.0248890.s008.docx]

**S5 Table.** Total number of upregulated genes/genotypes under different NaCl concentrations.

| 50 mM NaCl | | 150 mM NaCl | | 250 mM NaCl | |
| --- | --- | --- | --- | --- | --- |
| Sohag 5 | **0** | Misr 2 | **0** | Sohag 5 | **0** |
| Misr 1 | **1** | Misr 1 | **0** | Giza 186 | **0** |
| Giza 186 | **2** | Sids 1 | **0** | Gemmeiza 12 | **0** |
| Sakha 95 | **2** | Sohag 5 | **2** | Sids 12 | **0** |
| Beni suwif 7 | **3** | Giza 186 | **4** | Sids 1 | **0** |
| Gemmeiza 12 | **4** | Sohag 4 | **4** | Sakha 95 | **0** |
| Sids 1 | **4** | Shandaweel 1 | **5** | Beni suwif 7 | **0** |
| Misr 3 | **5** | Sakha 95 | **5** | Sohag 4 | **0** |
| Misr 2 | **6** | Sids 14 | **6** | Sakha 93 | **0** |
| Shandaweel 1 | **6** | Beni suwif 7 | **6** | Misr 2 | **1** |
| Sids 14 | **6** | Gemmeiza 12 | **7** | Misr 1 | **1** |
| Sohag 4 | **6** | Sids 12 | **7** | Shandaweel 1 | **2** |
| Sids 12 | **7** | Misr 3 | **7** | Misr 3 | **3** |
| Sakha 93 | **7** | Sakha 93 | **8** | Sids 14 | **3** |
