# Supplementary material for: Detection of genetic divergence among some wheat (Triticum aestivum L.) genotypes using molecular and biochemical indicators under salinity stress
Source: PLoS One. 2021 Mar 29;16(3):e0248890. doi: 10.1371/journal.pone.0248890 (PMC8007010; doi:10.1371/journal.pone.0248890)

Fig. 5

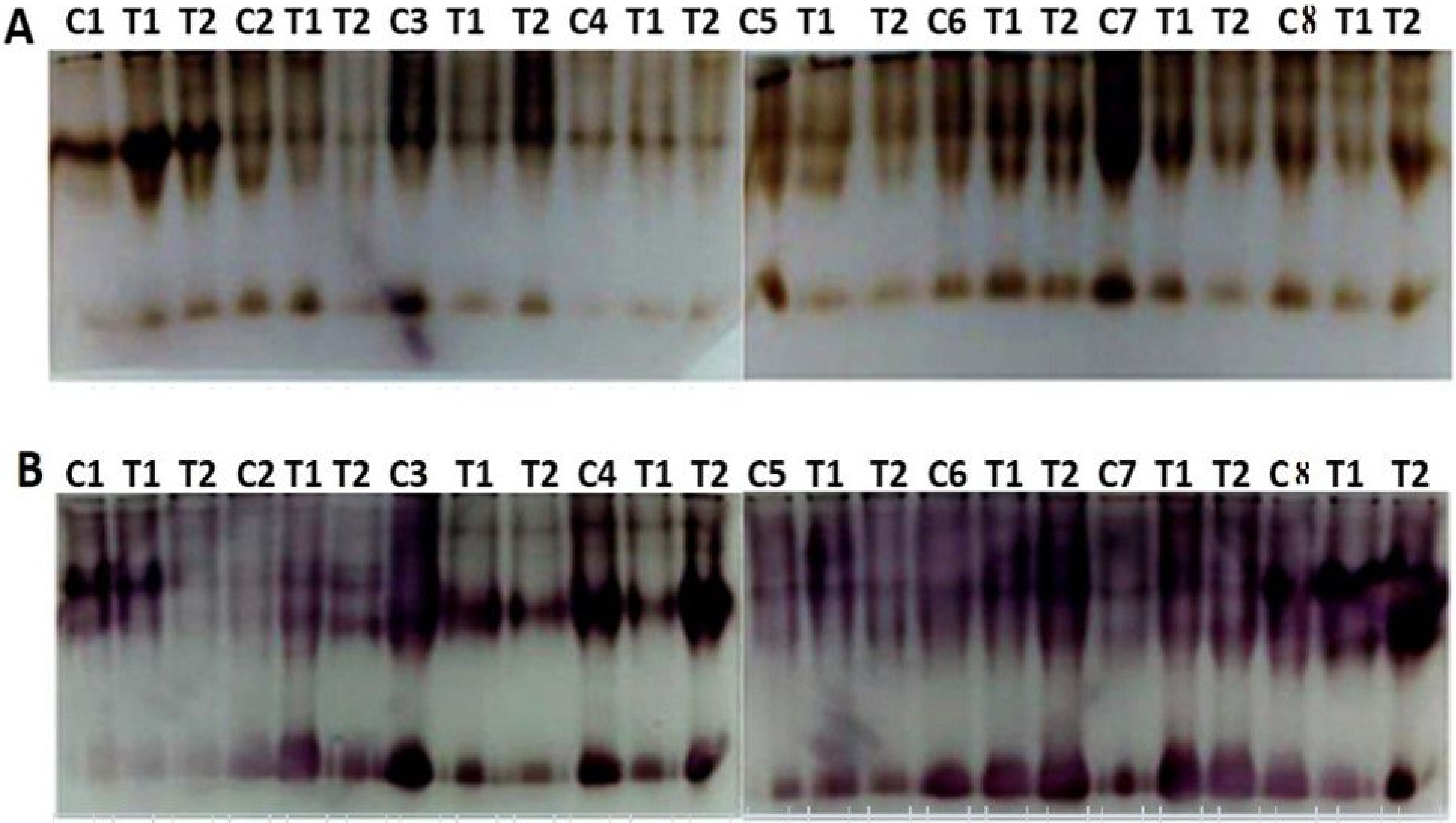
A
C1 T1 T2 C2 T1 T2 C3 T1 T2 C4 T1 T2 C5 T1 T2 C6 T1 T2 C7 T1 T2 C8 T1 T2
B
C1 T1 T2 C2 T1 T2 C3 T1 T2 C4 T1 T2 C5 T1 T2 C6 T1 T2 C7 T1 T2 C8 T1 T2

Figure 5 A left

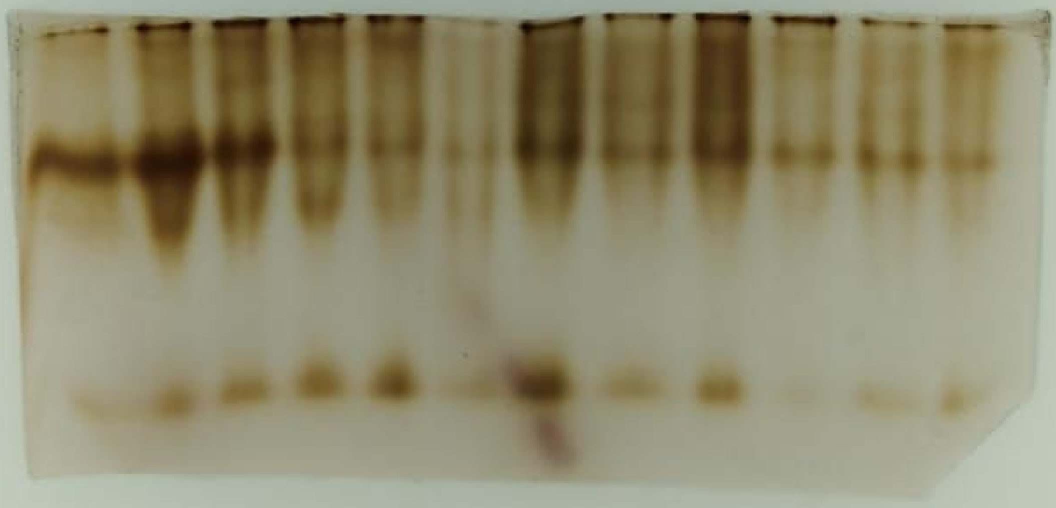

Figure 5 A right

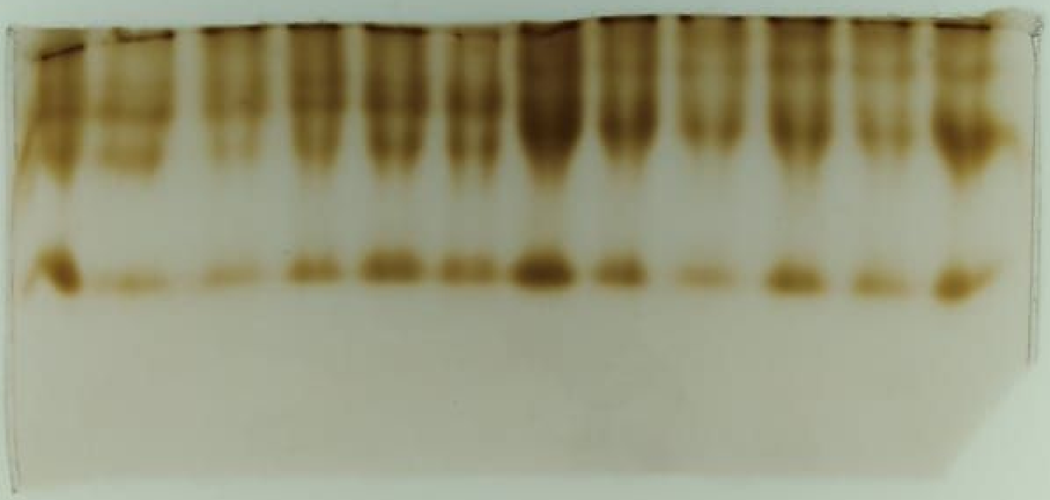

Figure 5 B left

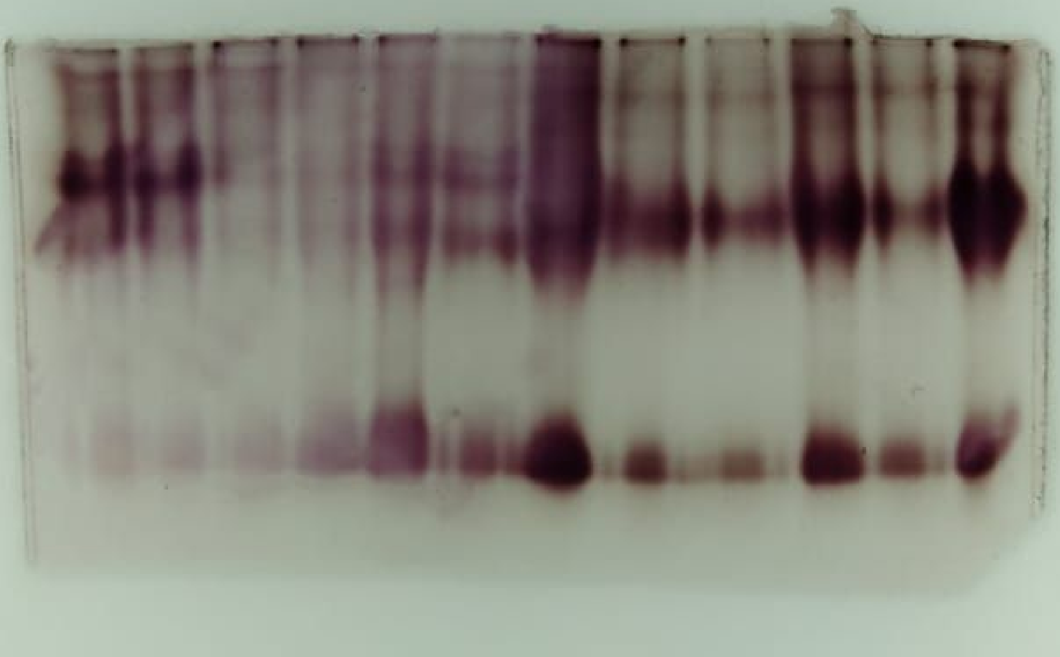

Figure 5 B right

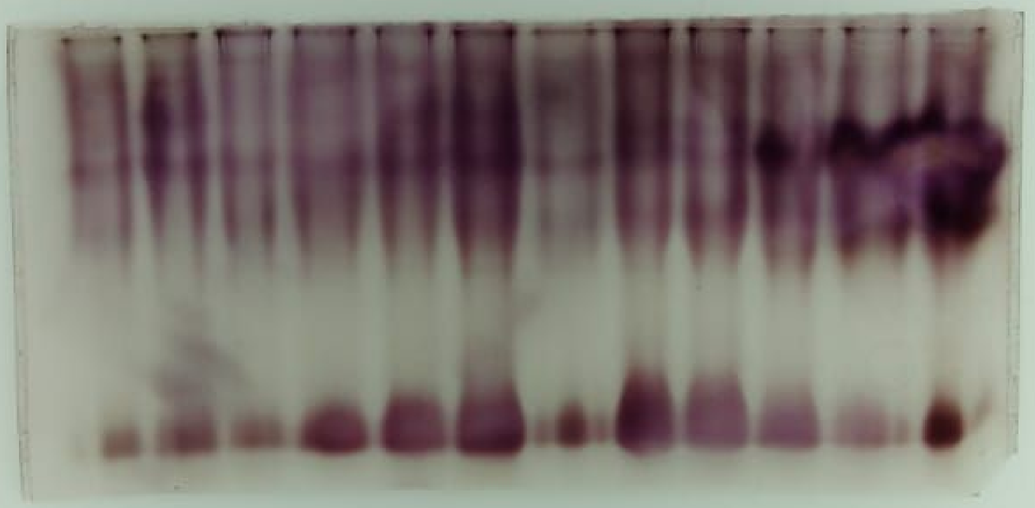

S1 Fig.

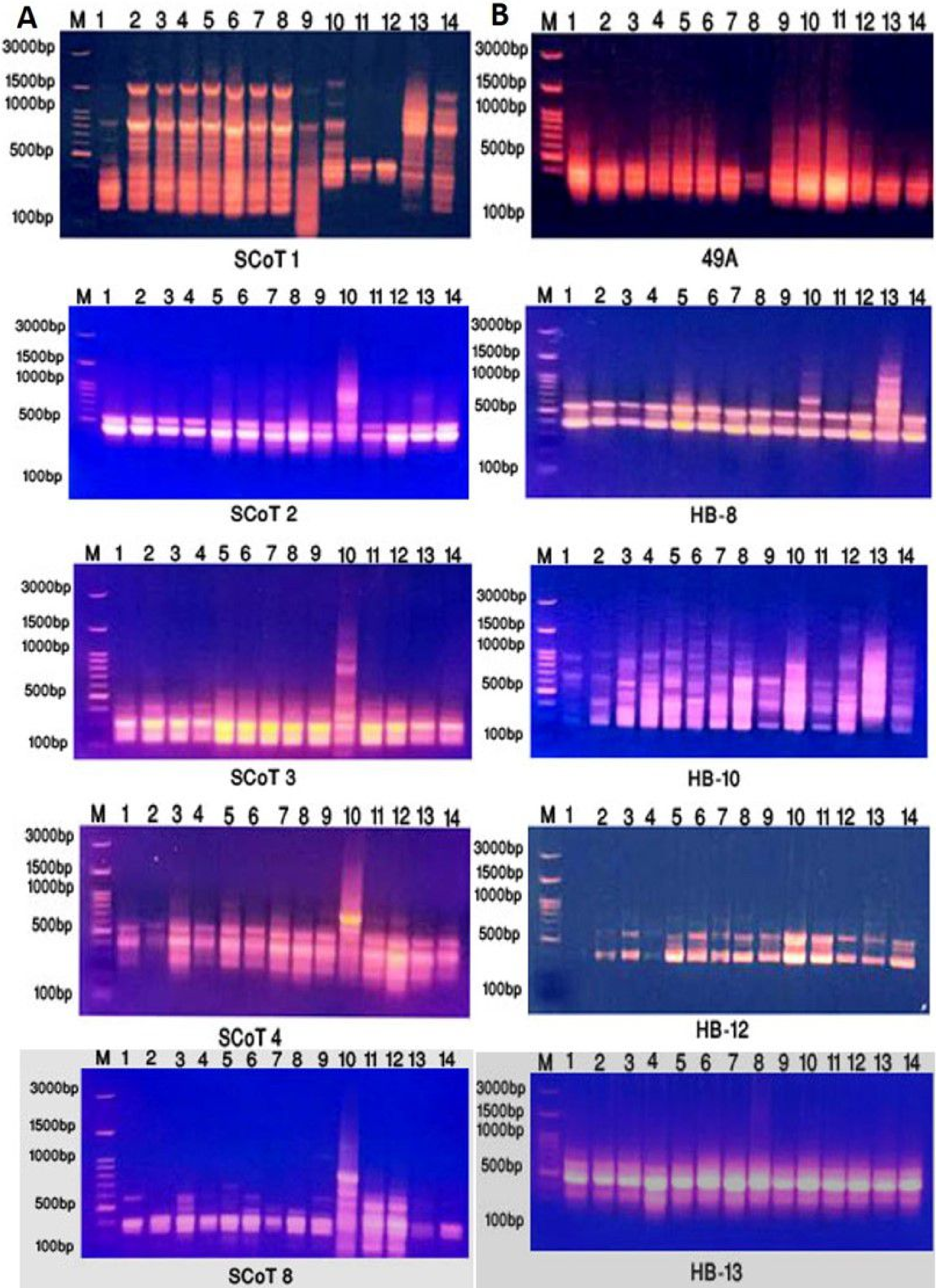
A
B
M 1 2 3 4 5 6 7 8 9 10 11 12 13 14
3000bp
1500bp
1000bp
500bp
100bp
SCoT 1
49A
SCoT 2
HB-8
SCoT 3
HB-10
SCoT 4
HB-12
SCoT 8
HB-13

49 A

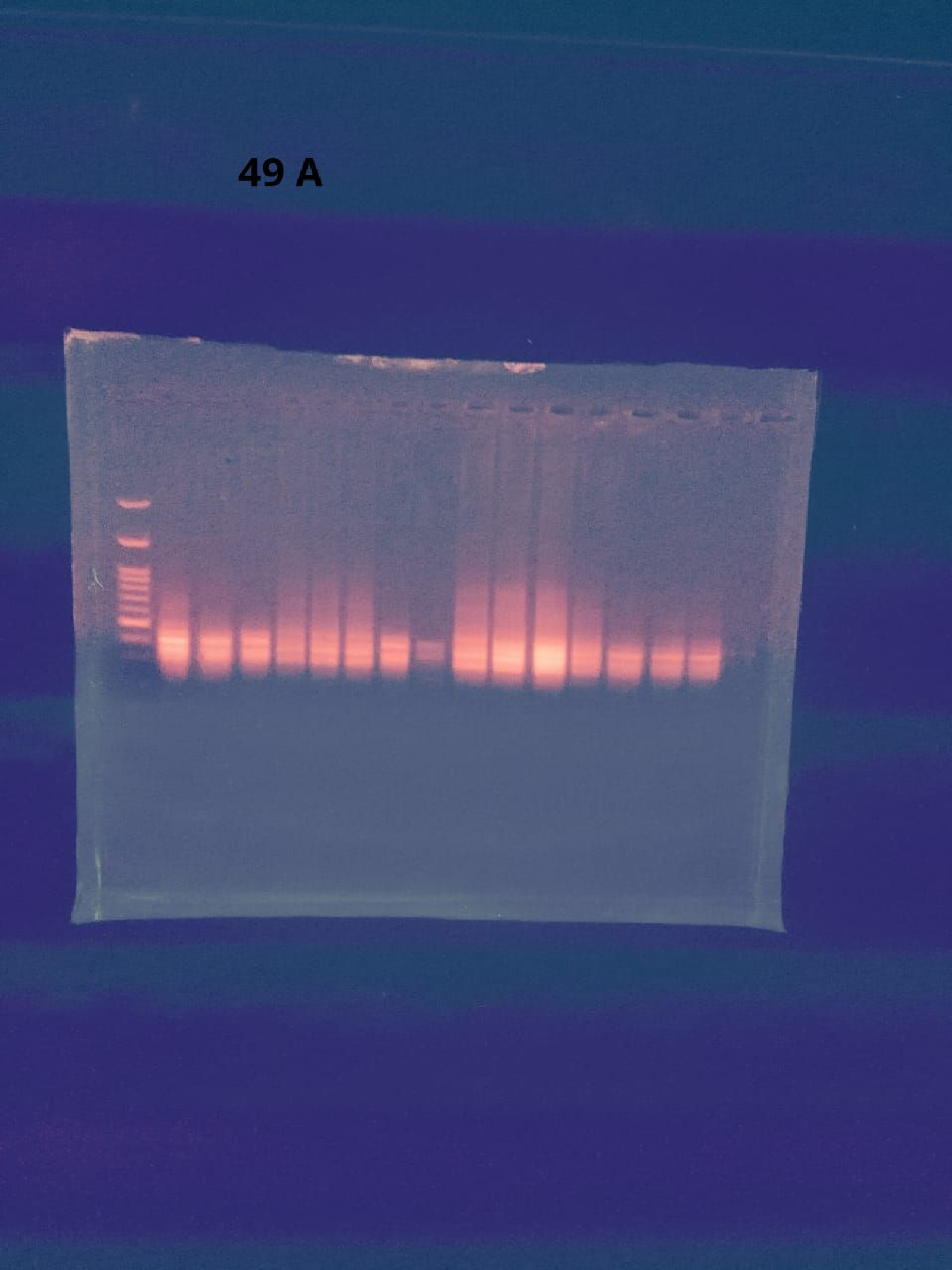

HB-08

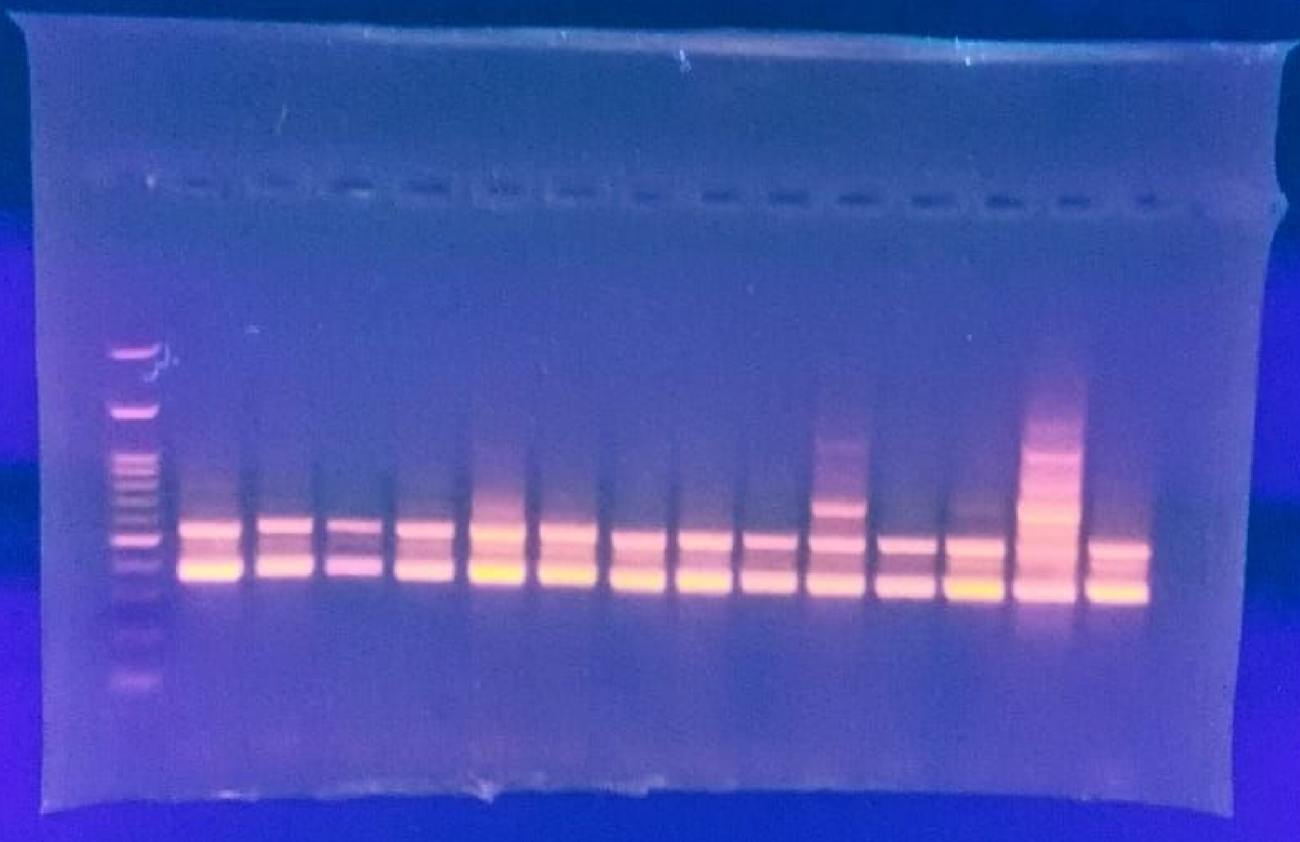

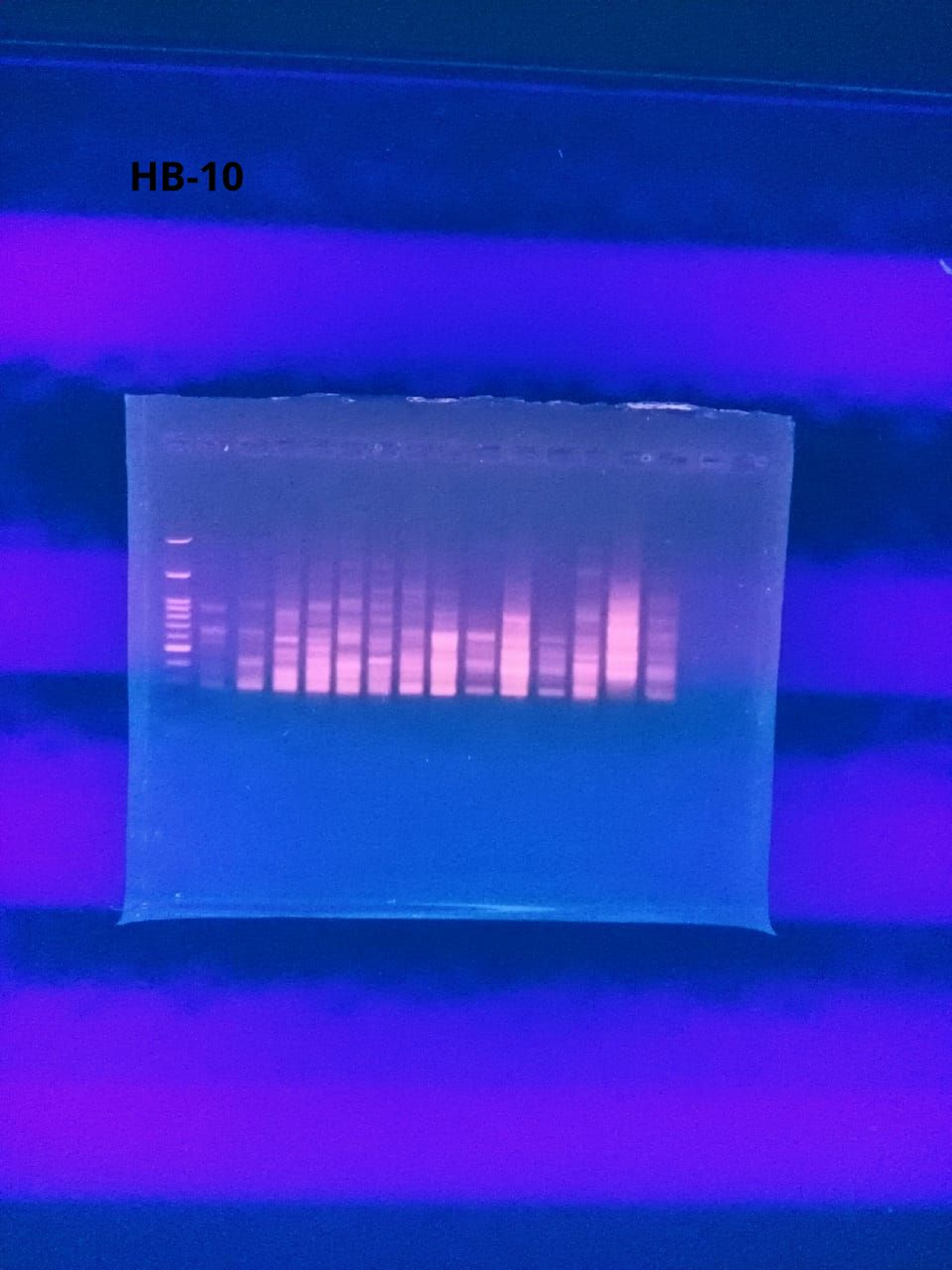
HB-10

HB-12

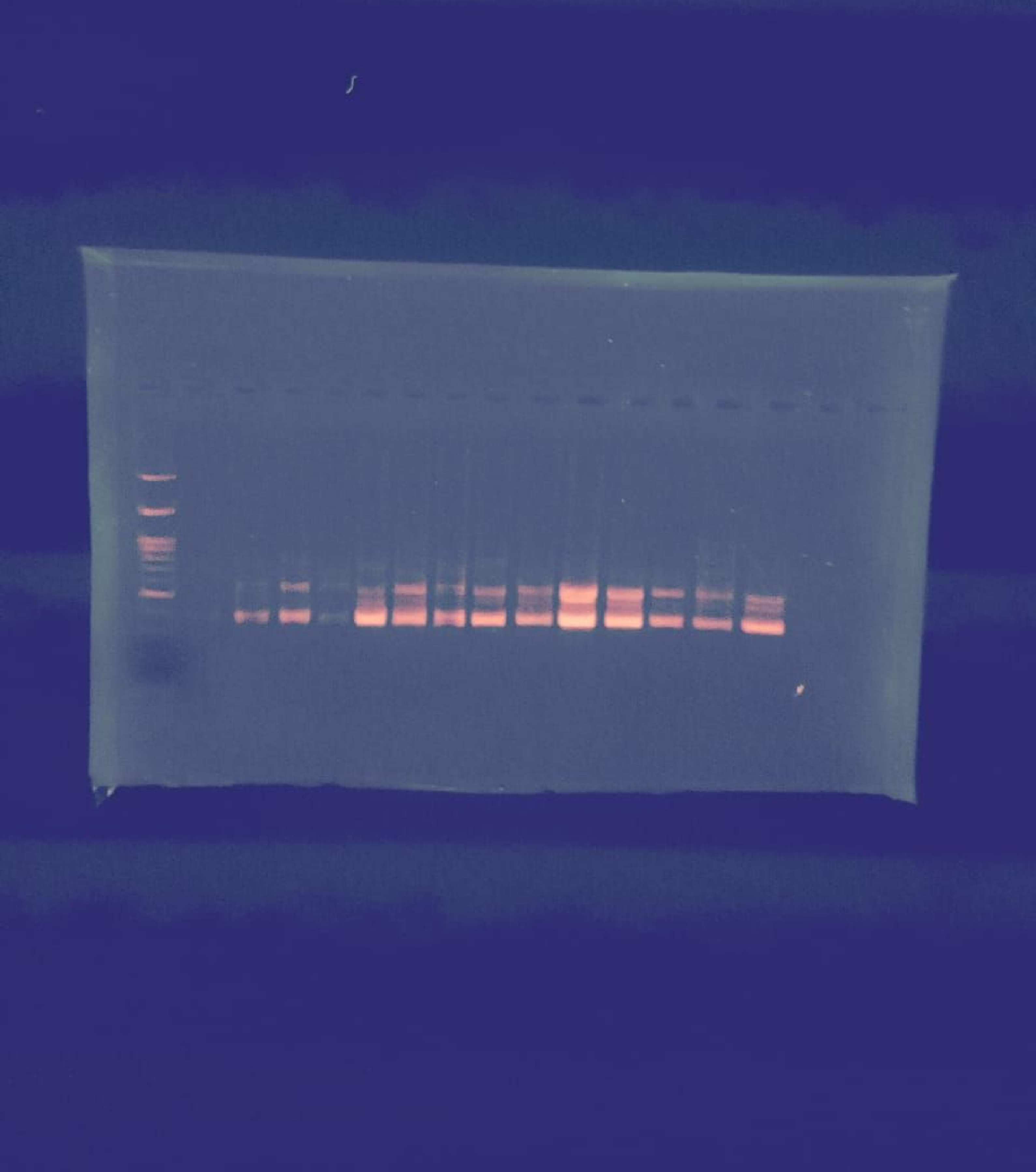

HB-13

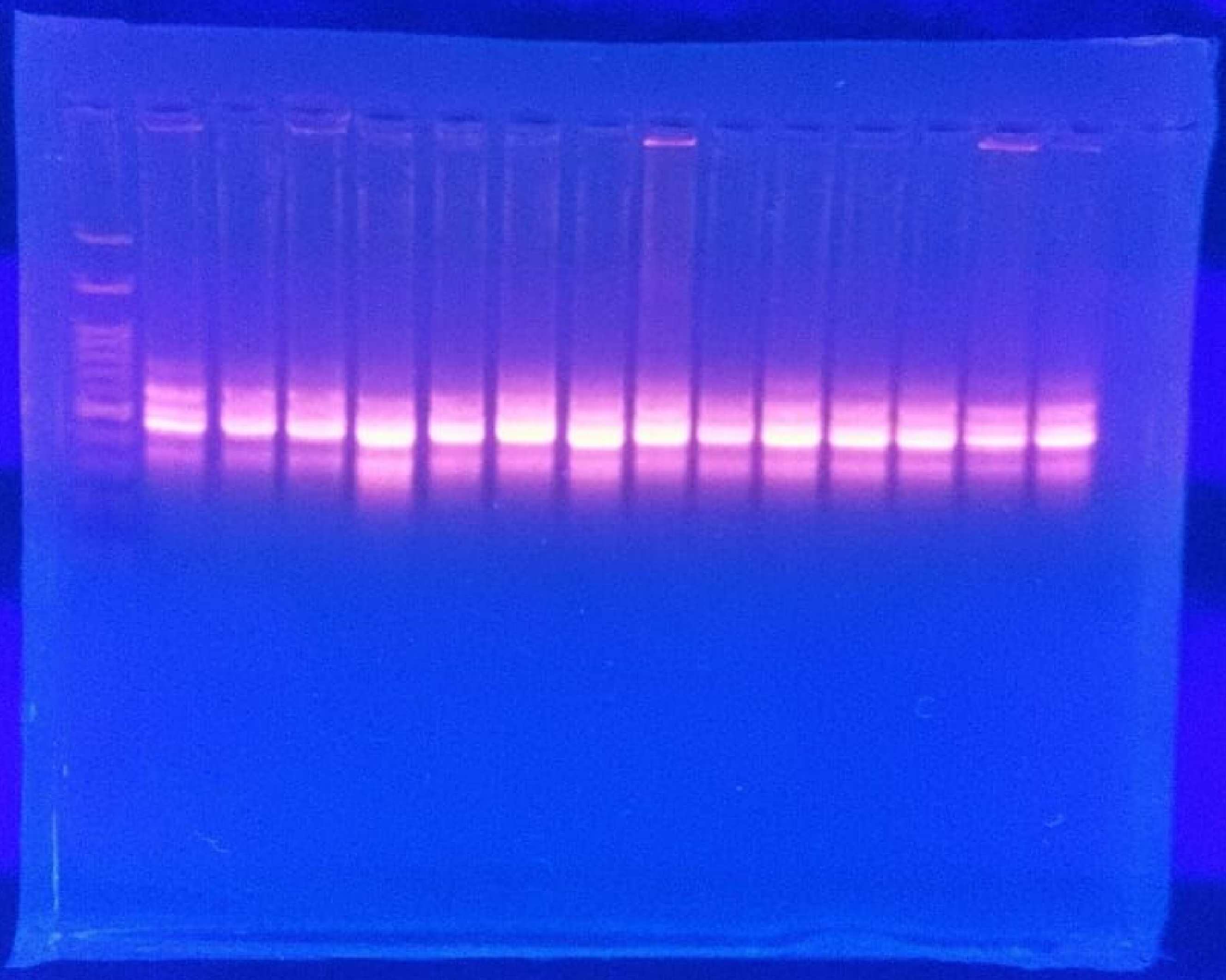

SCoT 1

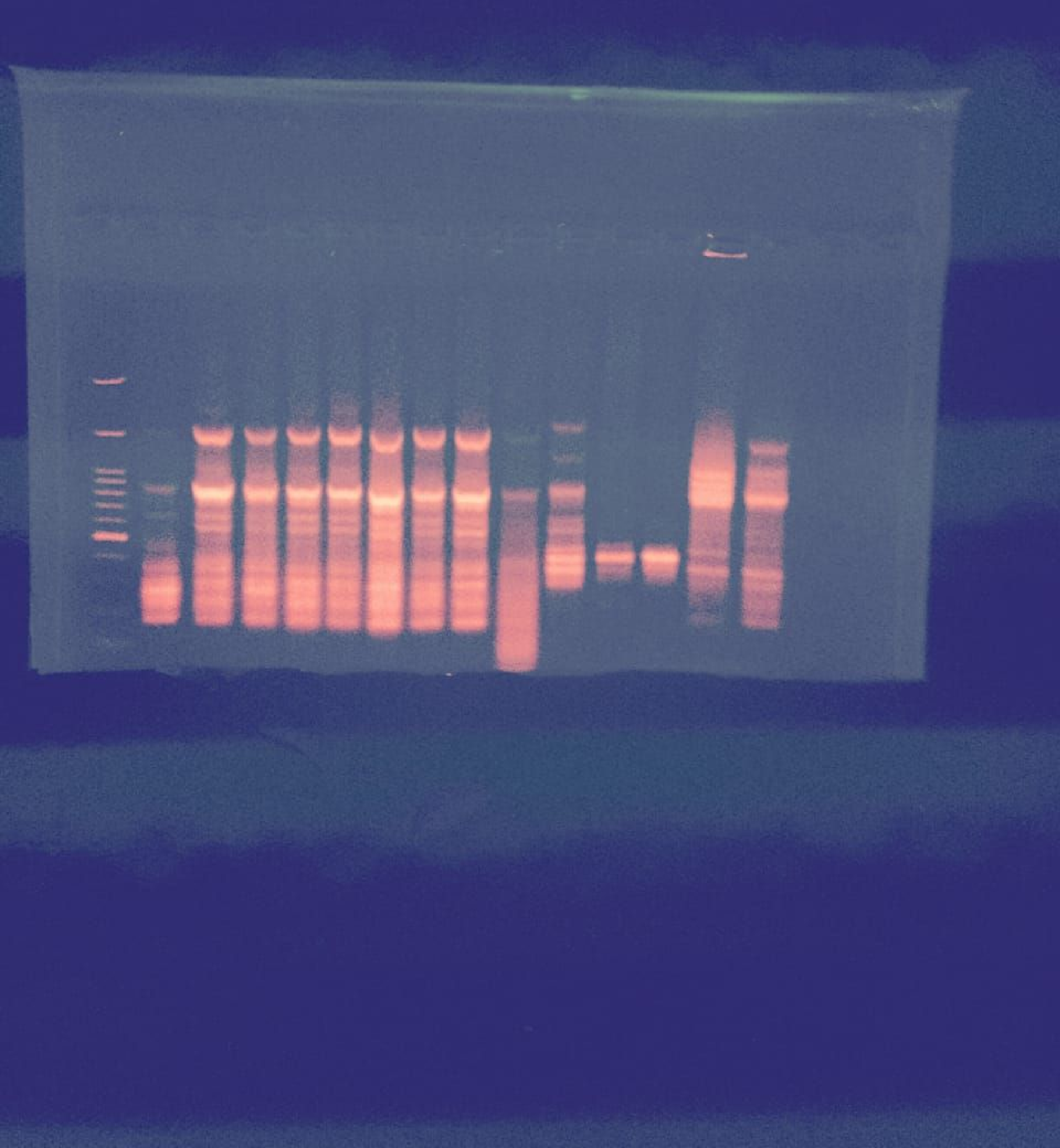

SCoT 2

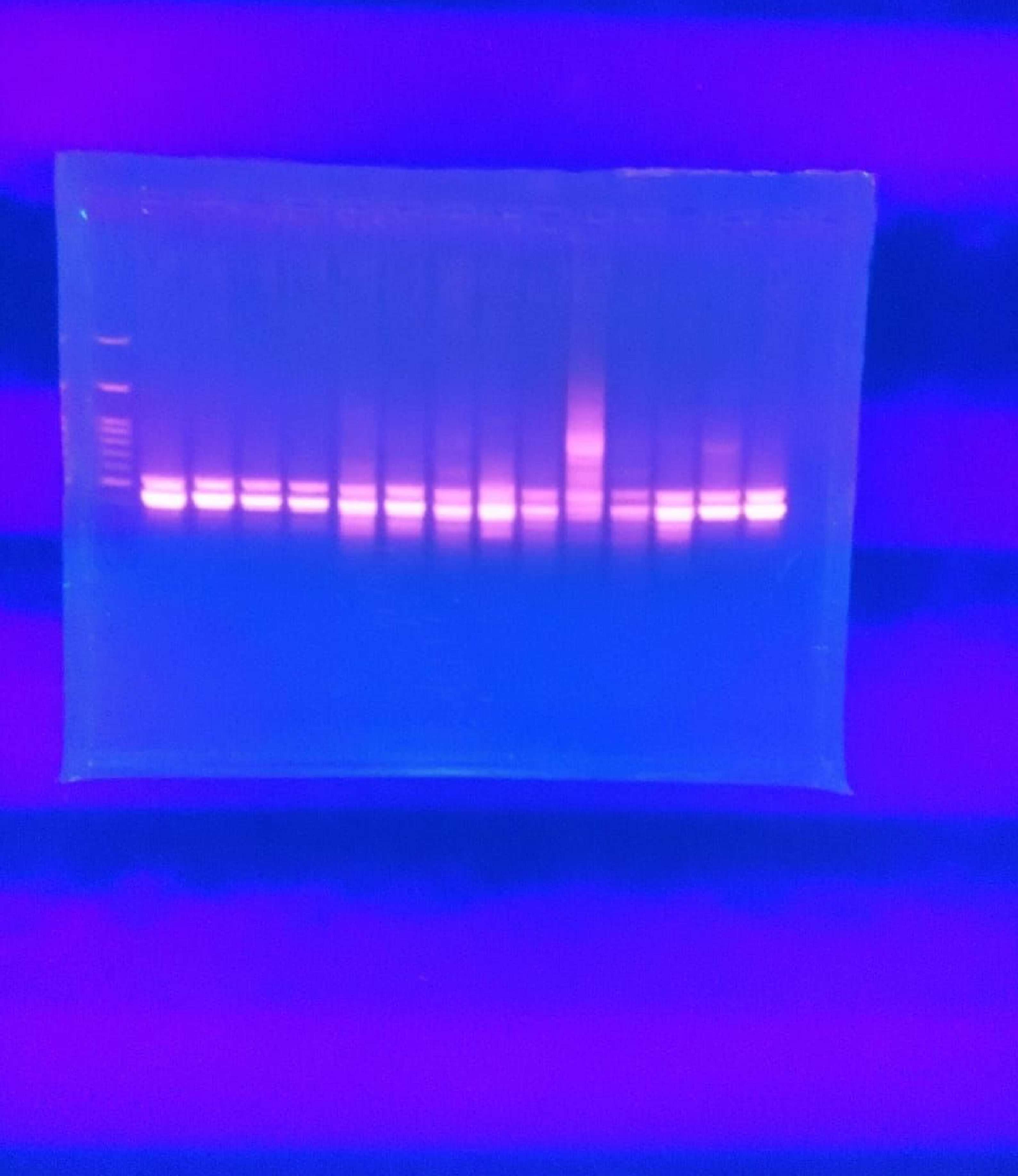

SCoT 3

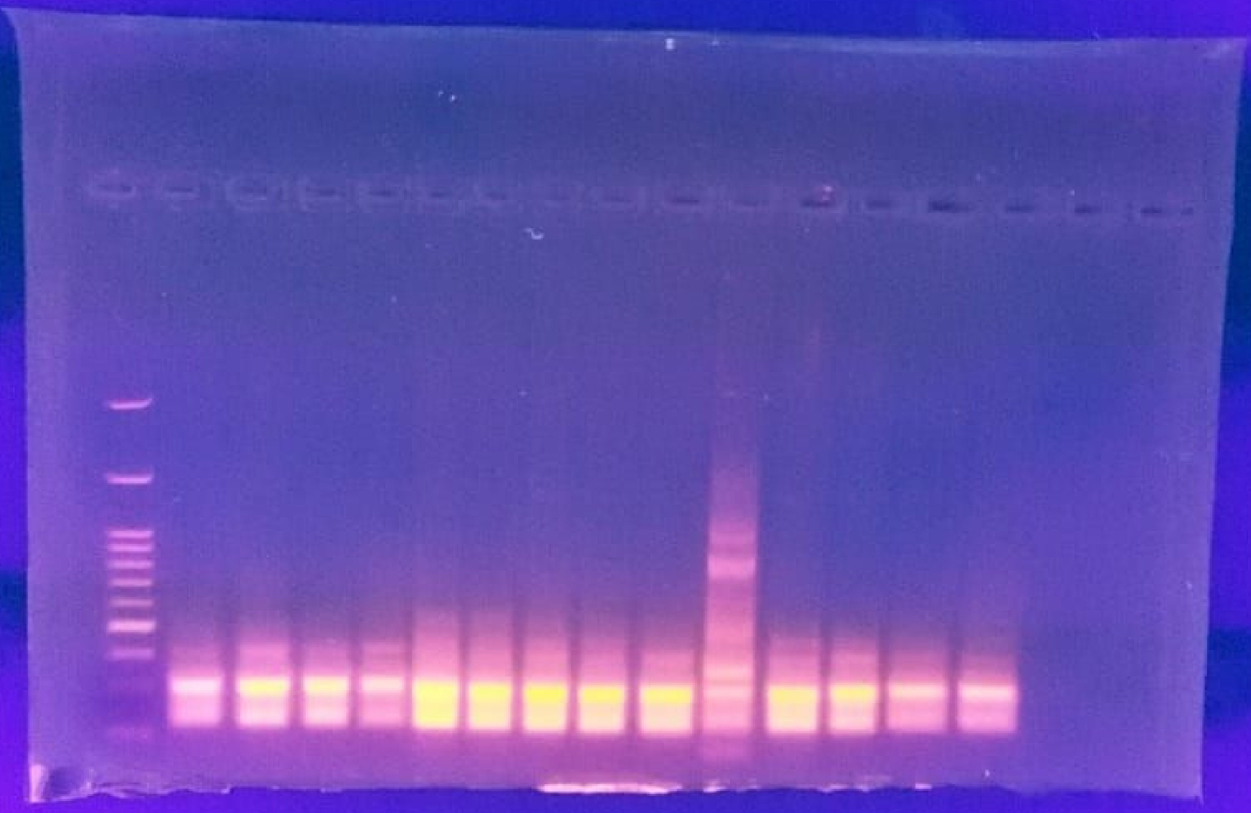

SCoT 4

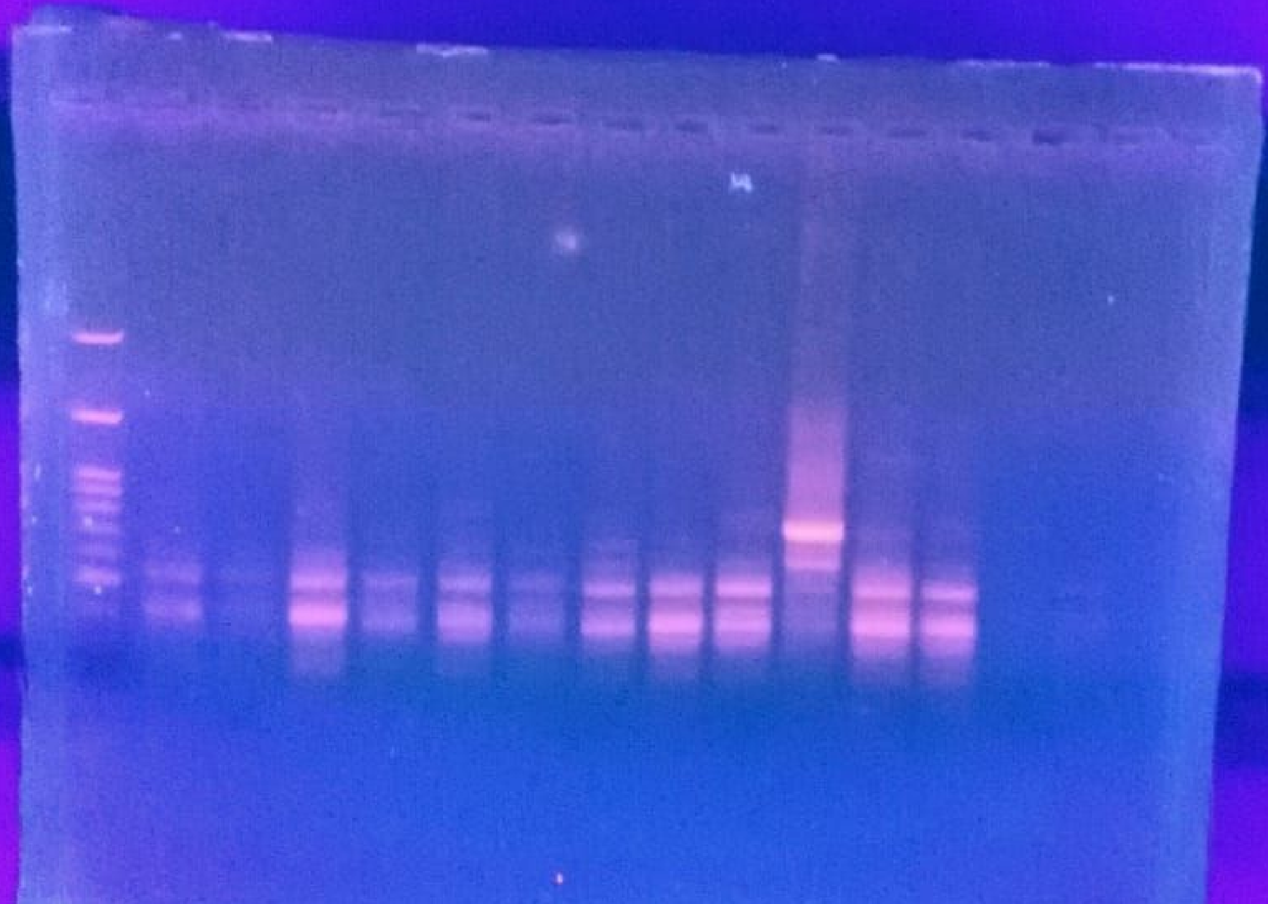

SCoT 8

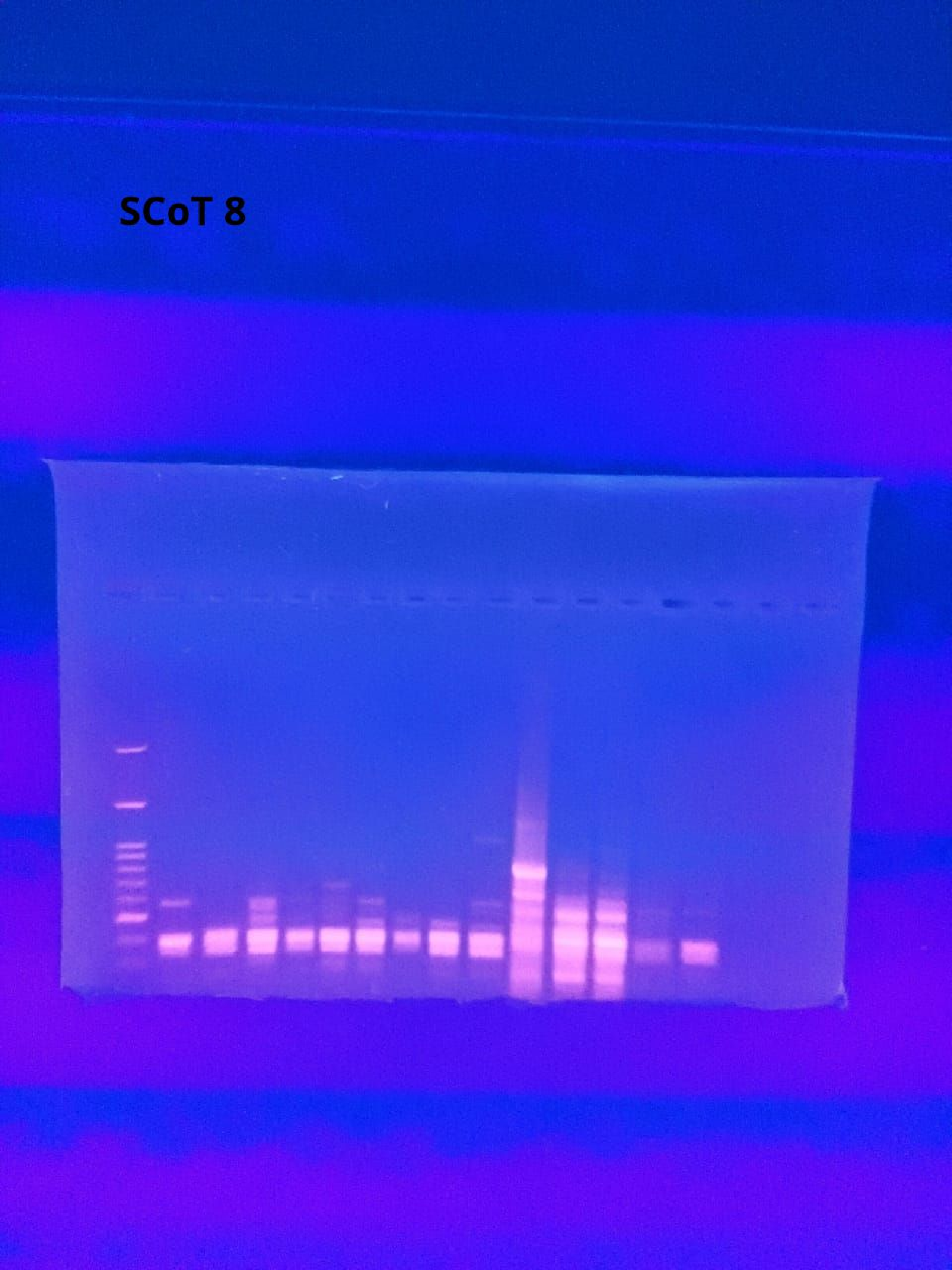

Supplement: S1 Raw image — (PDF) [file pone.0248890.s009.pdf]
