# Supplementary material for: Detection of genetic divergence among some wheat (Triticum aestivum L.) genotypes using molecular and biochemical indicators under salinity stress
Source: PLoS One. 2021 Mar 29;16(3):e0248890. doi: 10.1371/journal.pone.0248890 (PMC8007010; doi:10.1371/journal.pone.0248890)
Supplement: Attachment — Submitted filename: Response to Reviewers.docx [file pone.0248890.s011.docx]

Response to Reviewer 1 Comments

First, we are very appreciative of the great efforts of the reviewer in reviewing our manuscript, and we are very thankful for all the valuable comments and suggestions, which have improved our manuscript. In this regard, below we provide detailed answers to each of the requests and concerns raised by the reviewers. Moreover, we have attached a revised version of our manuscript that includes all the suggested corrections.

**Point 1:** Firstly, the formatting of the paper should be checked to ensure that they meet the requirements of the journal, the major issue being the tables and the headings.

**Response 1:** We have checked the formatting of the paper, and the revised manuscript as well as the tables and the figures follows the journal instructions.

**Point 2**: Secondly, mostly seen at the materials and methods section, references were made to numbers, which I believe is not in right order. For example, "seeds were sterilized and then germinated for three weeks using the method described by [28]". Please rectify.

**Response 2:** This has been corrected in the new revised manuscript file. Moreover, we have examined the entire manuscript to ensure that all the inserted references are numbered in the appropriate order.

**Point 3:** Thirdly, parameters describing the informativeness of the markers including average number of alleles per locus, average number of effective alleles, the mean heterozygosity per locus could be included.

**Response 3:**

- All the required parameters have been calculated, and the results are included in Table 1.
- Please note that we have reconstructed Table 1, which was two separate tables in the old version. In its new format, this table now includes all the estimated and calculated parameters from the old version of the manuscript (MB: monomorphic band, UB: unique band, PB: polymorphic band, TAB: total amplified bands, FS: fragment size, PIC: polymorphic information content, EMR: effective multiplex ratio, MI: marker index, P%: percent of polymorphism, Rp: resolving power) as well as all the new calculated parameters (Na: average number of alleles per loci, Ne: average number of effective alleles per loci, He: expected heterozygosity, uHe: unbiased expected heterozygosity).
- In addition, please note that it was difficult to calculate the mean heterozygosity per locus for the studied ISSR and SCoT primers. Because the amplification bands are often dominant markers, it is impossible to effectively identify homozygotes and heterozygotes (Isabel et al., 1999; McGregor et al., 2000 and Burgess 2001). Therefore, we calculated the expected heterozygosity and unbiased expected heterozygosity.
- Additionally, in the Material and Methods section, we have included the methods that were used to calculate the new parameters. The Results and Discussion have been updated accordingly.

**Point 4**: Fourthly, the discussion section is all put together, with no paragraphing makes reading difficult. Moreover, the explanation could be concise and better explaining.

**Response 4**: This has been corrected in the revised manuscript. Please note that the format of the discussion has been reconstructed in separate paragraphs as requested. Simultaneously, we have examined the entire Discussion and deleted some parts, in addition to revising others for clarity and concision.

**Point 5**: Finally, authors should scan through the entire manuscript for minor typographical and grammatical errors, as such.

**Response 5**: Before we submitted our paper for review by your respected journal, we had our manuscript edited for proper English language, grammar, punctuation, spelling, and overall style by one or more of the highly qualified native English speaking editors at AJE (please see the attached certificate). In addition, we have reedited the corrected manuscript using the same service (please see the attached certificate).

Response to Reviewer 2 Comments

**Point 1:** The paper is written in very well manner. Still need improvement in English. Overall, the paper is good and can be proceed further for evaluation.

**Response 1**: Before we submitted our paper for review by your respected journal, we had our manuscript edited for proper English language, grammar, punctuation, spelling, and overall style by one or more of the highly qualified native English speaking editors at AJE (please see the attached certificate). In addition, we have reedited the corrected manuscript using the same service (please see the attached certificate).
