# Supplementary material for: Detection of genetic divergence among some wheat (Triticum aestivum L.) genotypes using molecular and biochemical indicators under salinity stress
Source: PLoS One. 2021 Mar 29;16(3):e0248890. doi: 10.1371/journal.pone.0248890 (PMC8007010; doi:10.1371/journal.pone.0248890)
Supplement: Attachment — Submitted filename: Response to Reviewers.docx [file pone.0248890.s012.docx]

Response to Reviewer 1 Comments

First, we are very appreciative of the great efforts of the reviewer in reviewing our manuscript, and we are very thankful for all the valuable comments and suggestions, which have improved our manuscript. In this regard, below we provide detailed answers to each of the requests and concerns raised by the reviewers. Moreover, we have attached a revised version of our manuscript that includes all the suggested corrections.

**Point 1:** Manuscript is well written. However, authors should go through the formatting very well to ensure that they are consistent. Especially with the headings and sub-headings. In all, paper has been properly presented.

**Response 1:** thank you so much for your positive comment, we have checked the formatting of the paper, as well as the headings and sub-headings to be sure that all are in the suitable font.

Response to Reviewer 2 Comments

**Point 1**: Please check and review this paper again. Some grammatical mistakes are still present. Some minor mistakes of grammar.

**Response 1**: we have checked the entire manuscript to detect any grammatical mistakes. Please see the attached revised version that include all the corrections that we did.
